# Third Trimester Cerebellar Metabolite Concentrations are Decreased in Very Premature Infants with Structural Brain Injury

**DOI:** 10.1038/s41598-018-37203-4

**Published:** 2019-02-04

**Authors:** Sudeepta K. Basu, Subechhya Pradhan, Kushal Kapse, Robert McCarter, Jonathan Murnick, Taeun Chang, Catherine Limperopoulos

**Affiliations:** 1 Neonatology, Children’s National Health System, Washington, D.C. USA; 2 Developing Brain Research Laboratory, Children’s National Health System, Washington, D.C. USA; 3 Division of Bio-Statistics, Children’s National Health System, Washington, D.C. USA; 4 Division of Diagnostic Imaging and Radiology, Children’s National Health System, Washington, D.C. USA; 5 Division of Neurology, Children’s National Health System, Washington, D.C. USA; 6 The George Washington University School of Medicine, Washington, D.C. USA

## Abstract

Advanced neuroimaging techniques have improved our understanding of microstructural changes in the preterm supratentorial brain as well as the cerebellum and its association with impaired neurodevelopmental outcomes. However, the metabolic interrogation of the developing cerebellum during the early postnatal period after preterm birth remains largely unknown. Our study investigates the relationship between cerebellar neurometabolites measured by proton magnetic spectroscopy (^1^H-MRS) in preterm infants with advancing post-menstrual age (PMA) and brain injury during ex-utero third trimester prior to term equivalent age (TEA). We prospectively enrolled and acquired high quality ^1^H-MRS at median 33.0 (IQR 31.6–35.2) weeks PMA from a voxel placed in the cerebellum of 53 premature infants born at a median gestational age of 27.0 (IQR 25.0–29.0) weeks. ^1^H-MRS data were processed using LCModel software to calculate absolute metabolite concentrations of N-acetylaspartate (NAA), choline (Cho) and creatine (Cr). We noted positive correlations of cerebellar concentrations of NAA, Cho and Cr (Spearman correlations of 0.59, 0.64 and 0.52, respectively, p value < 0.0001) and negative correlation of Cho/Cr ratio (R −0.5, p value 0.0002) with advancing PMA. Moderate-to-severe cerebellar injury was noted on conventional magnetic resonance imaging (MRI) in 14 (26.4%) of the infants and were noted to have lower cerebellar NAA, Cho and Cr concentrations compared with those without injury (p value < 0.001). Several clinical complications of prematurity including necrotizing enterocolitis, systemic infections and bronchopulmonary dysplasia were associated with altered metabolite concentrations in the developing cerebellum. We report for the first time that ex-utero third trimester cerebellar metabolite concentrations are decreased in very preterm infants with moderate-to-severe structural cerebellar injury. We report increasing temporal trends of metabolite concentrations in the cerebellum with advancing PMA, which was impaired in infants with brain injury on MRI and may have early diagnostic and prognostic value in predicting neurodevelopmental outcomes in very preterm infants.

## Introduction

Cerebellar injury in preterm infants predicts poor cognitive and social-behavioral outcomes independent of supratentorial injury^1–5^. Very preterm infants born before 32 weeks gestational age (GA) are exposed to a hostile extra-uterine milieu during a period of very rapid cerebellar growth and maturation^6–9^. Cerebellar volume, sulcation depth and complex pattern of foliation dramatically increases during the second and third trimester^6,9^. Preterm birth is a major risk factor for cerebellar hemorrhagic injury and impaired volumetric growth of the cerebellum as demonstrated by conventional magnetic resonance imaging (MRI) in preterm infants at term equivalent age (TEA)^10–13^. Altered cerebellar connectivity has been reported even in the absence of gross structural injury on conventional MRI, suggesting the presence of subtle microstructural and functional injury below the threshold of detection by current standard imaging modalities^7,10,12,14,15^.

Proton magnetic resonance spectroscopy (^1^H-MRS) enables non-invasive measurement of the *in-vivo* concentrations of neurometabolites. Several studies have reported rapidly changing concentrations of metabolites in the cerebrum of preterm infants in postnatal life which possibly reflect its rapid growth and development^16–19^. Altered metabolite concentrations in the developing cerebrum on ^1^H-MRS have also been associated with poor neurodevelopmental outcomes and thus, may be an important prognostic biomarker for evolving brain injury^20,21^. However, knowledge of *in-vivo* cerebellar neurometabolites in preterm infants is limited to a few studies demonstrating altered cerebellar metabolic profile at TEA compared to healthy full-term infants and its association with impaired cognitive function at 2 years of corrected age^5,21–23^. The evolution of *in-vivo* metabolic profiles in the preterm cerebellum during the early ex-utero postnatal period prior to TEA remains largely unknown, which limits our understanding and capability to identify the evolving microstructural alterations that may guide timely intervention before brain injury is irreversibly consolidated.

In this study, we sought to characterize the cerebellar metabolic profiles with advancing postmenstrual age (PMA) prior to reaching TEA in very preterm infants by using ^1^H-MRS. We hypothesized that early preterm cerebellar metabolite concentrations would be altered in the presence of structural brain injury on conventional neuroimaging and clinical complications of prematurity.

## Methods

### Participants

Very premature infants born ≤32 weeks and of ≤1500 g birth weight, admitted between 2012 and 2016 to the neonatal intensive care unit (NICU) at Children’s National Medical Center (Washington, D.C.) were prospectively enrolled. Infants with congenital malformations or dysmorphic features suggestive of a genetic syndrome, confirmed metabolic disorder, proven perinatal central nervous system infection or chromosomal abnormality were excluded. Demographic, perinatal and postnatal clinical data were collected through medical records review and parental questionnaires. The study was approved by the Children’s National Medical Center Institutional Review Board and conducted in accordance with relevant guidelines and regulations. Written parental consent was obtained from the parent(s) of each participant.

### MRI and ^1^H-MRS acquisition

Enrolled infants underwent an early postnatal MRI when medically stable before 37 weeks completed PMA and another MRI after reaching TEA (PMA window of 38–41 weeks). For this manuscript, we report findings only from the ^1^H-MRS scans performed before 37 weeks corrected PMA on a 1.5 Tesla MRI scanner (Discovery MR450, General Electric Medical Systems, Waukesha, WI). The findings from TEA ^1^H-MRS (3 Tesla magnetic field strength) have been published separately, since the higher magnetic field influences measured metabolite concentrations and thus interfere with comparative interpretation^22,24^. MRIs were performed during natural sleep using a feed and swaddle technique and unless clinically indicated, sedatives or intravenous injection of contrast agents were not administered during the MRI. Preterm infants requiring temperature monitoring were scanned using a MRI-compatible incubator (LMT Medical Systems GmbH, Luebeck, Germany) and LMT single channel incubator transmit-receive head coil. As part of our multimodal MRI acquisition protocol, Single Shot Fast Spin Echo images (2-mm slice thickness, 0-mm gap, echo time TE) = 160 ms, repetition time (TR) = minimum) each in axial, coronal and sagittal planes and ^1^H-MRS were acquired towards the end of the protocol.

**Figure 1:**
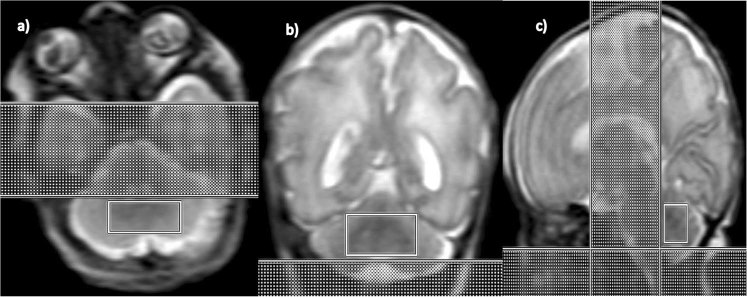
^1^H-MRS voxel placement in the preterm cerebellum: (**a**) Axial, (**b**) Coronal and (**c**) Sagittal views.

^1^H-MRS were acquired from a single voxel of 3 cm^3^ volume on average placed in the middle of the cerebellum avoiding the skull and extra-axial spaces (Fig. 1), using a point resolved spectroscopy (PRESS) sequence with TE = 144 ms, TR = 1500 ms, and 128 signal averages. Two oblique outer volume suppression bands were placed (Fig. 1) to minimize unwanted signals from outside the voxel and flow artifacts from the vasculature and cerebro-spinal fluid (CSF).

### Brain Injury Classification from Structural MRI

Each brain MRI study at TEA was reviewed and scored using Kidokoro *et al*.’s (2013) scoring system by an experienced pediatric neuroradiologist (JM) to identify the presence of moderate-to-severe cerebellar injury (overall score of ≥2 for either cerebellar signal abnormality or volume reduction) and overall brain injury (overall score of ≥8)^25^. The Kidokoro scoring system accounts for both white and gray matter signal and volume abnormalities in the cerebrum as well as cerebellum which are recognized as important markers of brain injury in preterm infants^25^. Germinal matrix hemorrhage and intraventricular hemorrhage (IVH) on routine serial cranial ultrasound were graded according to Papile’s grading system; and infants with grade ≥3 were classified as moderate to severe IVH^26,27^.

### ^1^H-MRS Data Pre- and Post-processing

**Figure 2:**
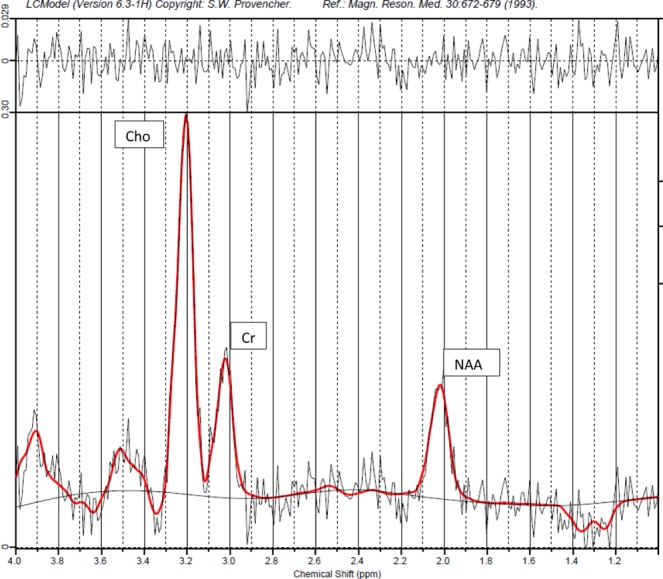
Representative ^1^H-MRS LCModel spectral output in a preterm infant.

^1^H-MRS data were processed using LCModel software (Provencher, 2001) to calculate metabolite concentrations in the chemical shift range of 4.0 to 1.0 ppm, using the unsuppressed water signal as an internal ref.^28^. An example of a spectrum with fit results from LCModel is presented in Fig. 2. The spectra generated by LCModel output were visually screened and additionally, spectra with either full width at line maximum (FWHM) greater than 0.172 ppm or a signal to noise ratio (SNR) of less than two were excluded. We have reported all metabolite concentrations in institutional units (i.u.) which represent metabolite concentrations derived using water as an internal reference^29^. The sums of metabolite concentrations for NAA (N-acetylaspartate + N-acetyl-aspartyl-glutamate), Cho (glycerophosphorylcholine + phosphocholine), Cr (creatine + phosphocreatine) were used in the analysis since they represent more reliable estimates. Individual metabolite concentrations of Lactate (Lac) and inositol (Ins) were also calculated. Metabolite concentrations with confidence levels greater than 20% of the Cramer-Rao lower bounds were excluded from subsequent analysis^30^. Given the overall low Lac levels in the preterm brain, only 2 infants had uncertainty estimates <20%. To obtain a more representative data sample for Lac, we relaxed our criteria to <40% and reported as Lac40^31,32^.

### Statistical analysis

Descriptive analyses compared means and frequencies of demographic and clinical characteristics in term and pre-term infants using Mann-Whitney Rank Sum test and chi square analyses, respectively. Spearman correlations were estimated between ^1^H-MRS metabolite concentrations/ratios and post-menstrual age at the time of imaging (PMA at MRI). Metabolite concentrations and ratios were compared between term and preterm infants, as well as, in infants with or without moderate-to-severe structural cerebellar injury on MRI (Mann-Whitney Rank Sum test). For bivariate associations with threshold P value <0.1, multiple quantile regression models were generated adjusting for GA, mode of delivery, maternal parity, child’s gender and race, PMA at MRI and presence of moderate-to-severe cerebellar injury on MRI. We developed statistical models to estimate the levels of neurochemical biomarkers at gestational age equivalents throughout this range while accounting for demographic differences in enrolled infants and standard errors used in statistical testing for the correlation between assessments in the same infant. Associations of the metabolite concentrations and ratios with clinical complications of prematurity [including definite necrotizing enterocolitis (NEC, Bell’s stage 2 or more), patent ductus arteriosus (PDA) ligation, bronchopulmonary dysplasia (BPD, moderate-to-severe by NICHD criteria), culture positive infection (CPI, blood stream or urinary tract), NEC and/or culture positive infection (NEC/CPI) and retinopathy of prematurity (ROP, stage 2 or higher in worse eye)] were investigated with multivariate regression adjusted for child’s GA, weeks of life (WOL) at MRI, gender and race. Statistical tests were conducted at a 2-tailed alpha level of 0.05. A P level of <0.05 was considered statistically significant. Statistical analyses were performed using SigmaPlot v14.0 (Systat Software Inc., San Jose, CA) and Stata release 14 (StataCorp LLC, College Station, TX).

## Results

### Descriptive Characteristics of our cohort

Cerebellar ^1^H-MRS from 53 infants born at a median GA of 27.0 (IQR 25.0–29.0; range 22.3–32) weeks met quality assessment criteria (63% out of 84 infants) for spectra acquired at a median 33.0 (IQR 31.6–35.2; range of 27.1–36.7) weeks PMA, and were included for this analysis. The median SNR in our overall cohort was 4.0 (IQR 3.0–6.0) and was comparable among infants with or without brain injury (P value 0.7). Demographic and clinical characteristics are presented in Table 1 and stratified by the presence of moderate-to-severe cerebellar injury.

**Table 1:** Baseline characteristics of preterm infants stratified by presence of moderate-to-severe cerebellar injury on conventional MRI.

Demographics	All infants with valid H^1^-MRS (n = 53)	Infants with no/mild cerebellar injury (n = 39)	Infants with moderate-to-severe structural cerebellar injury (n = 14)	P value
Birth Weight (in kg), median (IQR)	0.90 (0.65–1.17)	1.08 (0.73–1.28)	0.65 (0.58–0.87)	0.004
Gestational age (in weeks), median (IQR)	27.0 (25.0–29.0)	28.0 (25.9–29.6)	24.3 (23.1–26.1)	<0.001
WOL at MRI (in weeks), median (IQR)	5.7 (4.2–8.1)	5.4 (3.9–7.9)	7.8 94.9–9.2)	0.10
PMA at MRI (in weeks), median (IQR)	33.0 (31.6–35.2)	33.7 (31.6–35.9)	32.2 (30.7–33.3)	0.059
Female gender (%)	30 (56.6%)	22 (56.4%)	8 (57.1%)	0.8
Apgar at 5 min	10 (19.3%)	8 (6–9)	6 (4–8)	0.02
Gravida	3 (1–5)	2 (1–4)	3 (2–5)	0.2
Parity	1 (1–3)	2 (1–3)	1 (0–1)	0.035
Single gestation	41 (87.3%)	31 (79.5%)	10 (71.4%)	0.7
Child Race (White)	29 (54.7%)	23 (59.0%)	6 (42.9%)	0.4
Chorioamnionitis	12 (29.3%)	10 (25.6%)	2 (14.3%)	0.5
C-sec delivery	36 (67.9%)	30 (76.9%)	6 (42.8%)	0.042
Pregnancy induced hypertension	12 (22.6%)	10 (25.6%)	2 (14.3%)	0.5
Maternal diabetes	2 (3.8%)	1 (2.6%)	1 (7.1%)	0.5
Complications of prematurity				
BPD (moderate-to-severe)	27 (51.9%)	16 (41.1%)	11 (78.6%)	0.043
PDA ligation	10 (18.9%)	5 (12.8%)	5 (35.7%)	0.1
Definite NEC ≥ stage2	15 (28.3%)	9 (23.1%)	6 (40.0%)	0.2
NEC surgery	9 (17.0%)	5 (12.8%)	4 (28.6%)	0.2
Culture positive infection	10 (18.9%)	7 (17.9%)	3 (21.4%)	1
ROP ≥ stage 2	18 (34.6%)	10 (26.3%)	8 (57.1%)	0.052
IVH ≥ grade 3	14 (26.5%)	7 (17.9%)	7 (50.0%)	0.033
Brain injury (moderate-to-severe)	14 (26.5%)	13 (33.3%)	1 (7.1%)	<0.001

Demographic characteristics of infants included in this analysis compared with infants excluded for poor quality of spectra were similar (data not shown). Moderate-to-severe cerebellar injury was noted in 14 (26.4%) of the infants, out of which 7 (50%) infants had co-existing moderate-to-severe IVH. Infants with cerebellar injury were more likely to be younger and smaller at birth, born by cesarean section delivery, have lower Apgar scores at 5 minutes of life and were more likely to have co-existing complications of prematurity like moderate-to-severe BPD, IVH and ROP.

### Relationship of cerebellar metabolite concentrations with advancing PMA at MRI

Metabolite concentrations of NAA, Cho and Cr demonstrated significant bivariate positive correlation amongst each other (Spearman correlation R ~0.8) as well as advancing PMA at MRI (0.62, 0.54 and 0.63 respectively) with (Table 2).

**Table 2:** Relationship of cerebellar metabolite concentrations and ratios with PMA at MRI.

Metabolite/Ratio	No of infants with Valid H^1^-MRS	Mean ± SD (in i.u.)	Median (IQR) (in i.u.)	Correlation with PMA at MRI Spearman Rho (p value)
NAA	44	3.50 ± 1.31	3.24 (2.7–3.9)	0.59 (<0.0001)
Cr	52	4.57 ± 2.30	3.85 (3.1–5.5)	0.64 (<0.0001)
Cho	53	3.30 ± 1.06	3.15 (2.7–3.9)	0.51 (0.0001)
Ins	14	10.63 ± 7.02	16.36 (11.9–18.2)	−0.30 (0.3)
Lac40	21	1.77 ± 0.61	1.66 (1.9–2.1)	0.33 (0.1)
NAA/Cr	44	0.81 ± 0.23	0.78 (0.7–0.9)	−0.31 (0.04)
NAA/Cho	44	1.03 ± 0.20	1.03 (0.9–1.1)	0.33 (0.031)
Cho/Cr	53	0.79 ± 0.18	0.79 (0.7–0.9)	−0.5 (0.0002)
Ins/Cr	11	2.57 ± 1.37	4.67 (3.8–6.0)	−0.58 (0.03)
Lac40/Cr	18	0.45 ± 0.21	0.4 (0.3–0.5)	−0.43 (0.049)

Metabolite ratio for NAA/Cho had a significant positive correlation (R 0.32) whereas ratios of Cho/Cr, Lac40/Cr, and Ins/Cr demonstrated significant negative correlation (R −0.5, −0.43, −0.58 respectively) with advancing PMA at MRI. Relationships of NAA, Cr, Cho and Cho/Cr with advancing PMA at MRI remained statistically significant after adjusting for co-variates.

### Cerebellar metabolite concentrations were lower in presence of moderate-to-severe cerebellar injury on MRI

Infants with moderate-to-severe structural cerebellar injury on conventional TEA MRI had significantly lower cerebellar NAA (2.21 vs 3.44), Cr (3.0 vs 4.22) and Cho (2.46 vs 3.4) median concentrations at preterm ^1^H-MRS compared with infants without cerebellar injury (P value < 0.001, Table 3).

**Table 3:** Relationship of cerebellar metabolite concentrations and ratios with cerebellar injury on TEA MRI.

Metabolite Concentration or Ratio	No of infants with Cerebellar injury/Valid H^1^-MRS (n/N)	Infants with no/mild cerebellar injury Median (IQR) (in i.u.)	Infants with moderate-to-severe structural cerebellar injury Median (IQR) (in i.u.)	Mann-Whitney Test P value
NAA	11/44	3.44 (2.88–4.51)	2.21 (1.87–3.04)	<0.001
Cr	14/52	4.22 (3.50–6.37)	3.0 (2.22–3.5)	<0.001
Cho	14/53	3.4 (3.02–4.38)	2.46 (1.87–2.97)	<0.001
Ins	3/14	16.41 (11.62–17.5)	12.71 (11.97–20.37)	0.9
Lac40	6/21	1.72 (1.40–2.35)	1.51 (1.05–1.72)	0.09
NAA/Cr	11/44	0.78 (0.65–0.91)	0.76 (0.71–0.83)	1
NAA/Cho	11/44	1.04 (0.95–1.14)	0.88 (0.77–1.21)	0.3
Cho/Cr	14/53	0.78 (0.65–0.91)	0.85 (0.70–0.94)	0.4

**Figure 3:**
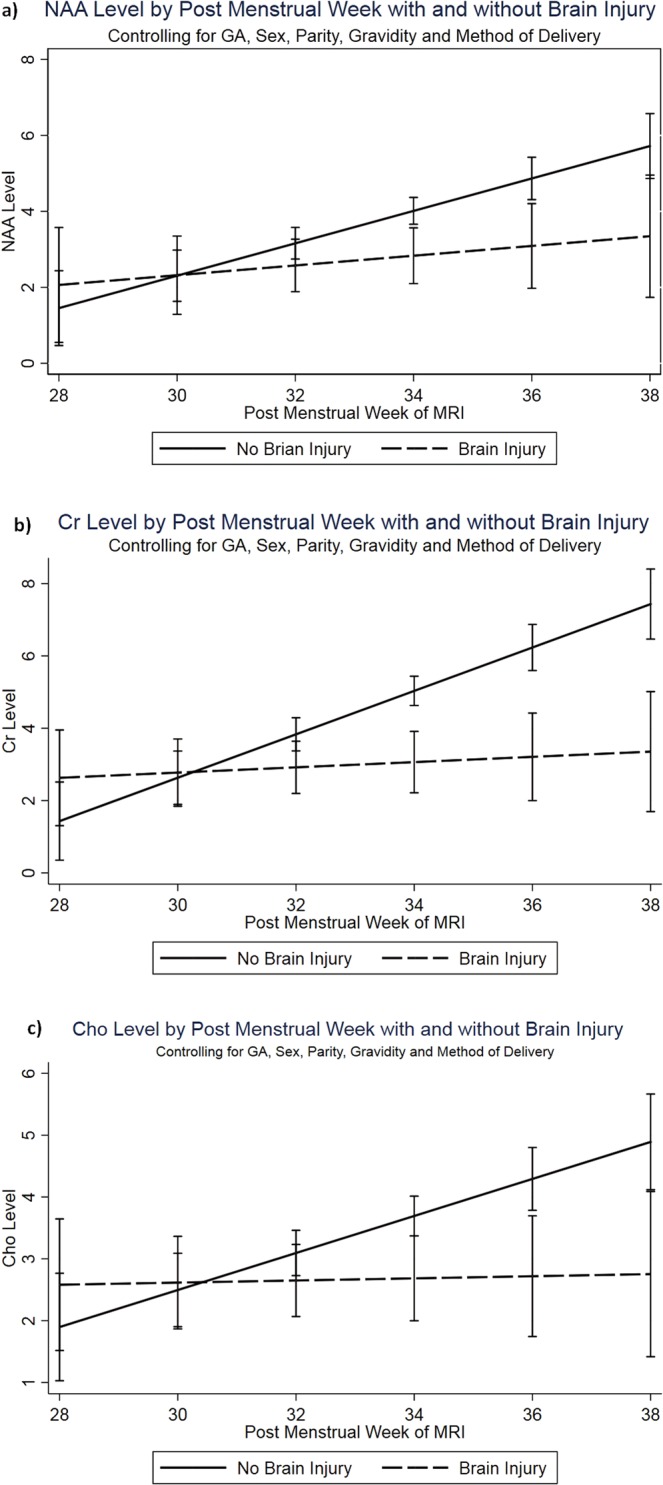
Metabolite profiles stratified by structural cerebellar injury on conventional MRI with advancing PMA at MRI: (**a**) NAA, (**b**) Cr and (**c**) Cho.

Using multivariate statistical models to estimate the levels of neurochemical biomarkers at gestational age equivalents throughout the range of 28–38 weeks corrected PMA, we observed that the association of decreased metabolites with moderate-to-severe brain (and cerebellar) injury remained statistically significant (Fig. 3).

### Relationship between cerebellar metabolites and clinical complications of prematurity

Bivariate and multivariate associations between clinical complications of prematurity and metabolites in the cerebellum are presented in Table 4. Infants with definite NEC stage ≥2 and/or culture positive infection (NEC ± CPI) had lower Cho, NAA, and Cr levels in the cerebellum. Other clinical complications of prematurity including PDA, BPD, ROP were associated with several cerebellar metabolite alterations as reported in Table 4.

**Table 4:** Association of clinical complications of prematurity with cerebellar metabolite concentrations and ratios.

Metabolites	Complications of prematurity	Infants without Clinical Complication Median (IQR) (in i.u.)	Infants with Clinical Complication Median (IQR) (in i.u.)	Mann-Whitney Test P value	Multivariate regression Coefficient (P value)
Cho	NEC ± CPI	3.35 (3.02–4.33)	2.65 (2.14–3.40)	0.002	−0.67 (0.025)
	BPD	3.4 (3.06–4.47)	2.98 (2.50–3.55)	0.021	−0.1 (0.7)
	NEC	3.30 (3.0–4.24)	2.65 (2.07–3.09)	0.003	−0.58 (0.011)
NAA	ROP	3.44 (3.04–3.96)	2.52 (1.96–3.54)	0.036	0.14 (0.7)
	NEC ± CPI	3.33 (2.86–4.22)	2.75 (2.01–3.72)	0.044	−0.46 (0.08)
	PDA lig	3.4 (2.82–3.96)	2.75 (2.05–3.33)	0.057	−0.08 (0.9)
	BPD	3.44 (2.93–4.94)	2.98 (2.24–3.70)	0.056	−0.41 (0.3)
Cr	NEC ± CPI	4.16 (3.45–6.05)	3.48 (2.72–4.59)	0.023	−0.72 (0.15)
	NEC	4.11 (3.30–5.92)	3.48 (2.79–3.90)	0.054	−0.65 (0.2)
	ROP	4.27 (3.48–5.92)	3.40 (2.83–3.95)	0.027	−0.15 (0.8)
NAA/Cho	NEC Surgery	1.02 (0.86–1.08)	1.21 (1.04–1.30)	0.030	0.21 (0.009)

*Multiple regression with co-variates in model: GA, WOL at MRI, Gender and Race.

## Discussion

We report for the first time, the ^1^H-MRS cerebellar metabolic profile in very premature infants during early postnatal period prior to reaching TEA and its relationship with brain injury and advancing PMA. We demonstrate significant positive correlations of cerebellar concentrations of NAA, Cho and Cr with advancing PMA adjusted for co-variates, perhaps reflecting rapid neuronal growth during early postnatal age. Infants with moderate-to-severe structural cerebellar injury had lower cerebellar NAA, Cho and Cr concentrations likely indicative of an altered metabolic milieu secondary to neuronal loss. Interestingly, we noted that infants with radiographic brain injury failed to demonstrate the temporal increase of neurometabolite concentrations in the cerebellum with advancing PMA. We postulate that this may reflect impaired or delayed neuronal growth compared to infants without structural brain injury. Several clinical complications of prematurity like NEC ± CPI, ROP, PDA and BPD were also associated with altered cerebellar metabolite concentrations.

One prior report of *in-vivo* cerebellar metabolites in preterm infants before reaching TEA was reported on a smaller group of 24 preterm infants by Tomiyasu *et al*., who noted a positive correlation of advancing PMA with myo-inositol and glutamate-glutamine complex but not for other metabolites^23^. Knowledge of *in-vivo* metabolites in preterm cerebellum at TEA is very limited as well. Brossard-Racine *et al*. previously reported altered cerebellar metabolite profile at TEA in VPT infants from our study cohort compared with term controls^22^. Van Kooij *et al*. reported association of cerebellar metabolites at TEA in VPT infants with impaired cognitive function at 2 years of corrected age^5^. Our study findings add to this knowledge as we report a statistically significant increase in NAA, Cho and Cr concentrations with advancing PMA. We also demonstrate that cerebellar biochemical maturation is impaired or delayed by structural brain injury on conventional neuroimaging and complications of prematurity. ^1^H-MRS studies on the cerebrum of preterm infants have previously reported increasing temporal trends of NAA, NAA/Cr and NAA/Cho with advancing age and its alterations on TEA ^1^H-MRS are better predictors of neurodevelopmental outcomes compared to structural MRI at TEA^16,18,19,31^. Given the limited *in-vivo* data available from the developing human cerebellum, our study findings are hypothesis generating. We postulate that the temporal increase in metabolite concentrations on cerebellar ^1^H-MRS may reflect the rapid postnatal neuro-axonal growth and its observed alterations in presence of brain injury may serve as a prognostic tool for neurodevelopmental outcomes. Long-term outcome studies are needed to confirm this.

The high collinearity observed among NAA, Cho and Cr potentially indicate a common underlying change in neuronal volume and density associated with advancing age and cerebellar injury prior to reaching TEA. NAA is an amino-acid synthesized primarily in neuronal mitochondria and plays a vital role in myelination, metabolism and modulation of neurons^33,34^. Cr is necessary for the regulation of energy supply in cells and together with NAA is considered to correlate with neuronal cell mass^34^. Consistent with this, our finding of a positive temporal correlation of NAA and Cr perhaps represents increasing neuronal proliferation and growth in the developing cerebellum. Cho is involved in membrane synthesis and typically decreases with myelination and maturation; whereas elevated levels could indicate the breakdown of myelin^34^. While ^1^H-MRS based animal studies and older human subjects have reported a stable to negative temporal trend in Cho concentrations^35–37^, recent studies have indicated a rapid increase in Cho concentrations in the developing cerebrum of preterm and term infants during early postnatal period^23,34^. Our observed positive trend in individual concentrations of Cho likely reflects the rapid neuro-axonal growth and turnover of cell membrane by-products during the late gestational and early postnatal period. Further, the comparative negative correlation of Cho/Cr likely may reflect developmental maturation of the white matter in the cerebellum with advancing PMA.

The association of infection with decreased cerebral NAA/Cho ratio has been previously described^38^. Animal models of systemic inflammation have been shown to lead to microstructural cerebellar injury^39^. In this cohort, we observed an independent association of systemic infection and NEC with several metabolic alterations in the developing cerebellum in the early postnatal period. Decreased white matter and cerebellar volumes on quantitative MRI has been reported in infants with any stage of ROP^40,41^ but MR spectroscopic alterations are unknown. In our study, infants with ROP (stage 2 or higher) had lower NAA and Cr concentrations compared to those without ROP although the relationship lost statistical significance after adjusting for co-variates. Whether there is a mechanistic relationship underlying these associations explaining the neurologic deficits associated with prematurity is an intriguing question that needs further investigation.

Although our study is strengthened by its prospective design, team expertise in acquiring non-sedated MRI in preterm population, the limitations deserve mention. Due to technical challenges of acquiring ^1^H-MRS reliably from a small developing cerebellum of preterm infants surrounded by posterior fossa structures and relative abundance of CSF in addition to motion-artifacts, only 53 (63%) preterm H^1^-MRS scans met our qualitative criteria for inclusion for analysis. We have included metabolite concentration data from ^1^H-MRS spectra with SNR as low as two as long as they met visual screening and <20% Cramer-Rao lower bounds criteria to allow maximal representation of the study population. Although we manually positioned each voxel to ensure that it predominantly contained cerebellar white matter, due to the shape and extensive foliation of the cerebellum, position of its deep nuclei and motion artifacts, our voxels likely contain a small portion of grey matter and CSF as well. The high degree of overlap between cerebellar injury and supratentorial injury on classification by Kidokoro scoring in our study cohort did not allow meaningful exploration of cerebellar injury related to diaschisis effect. Our relatively modest sample size might have limited our ability to detect more complex relationships with clinical risk factors and complications. The observed temporal relationship of metabolites with advancing PMA is limited to a single time point of pre-term scans (i.e., cross-sectional) and not based on true longitudinal observations for each infant. Finally, the lack of long-term neurodevelopmental outcomes is a limiting factor in interpreting clinical implications of our findings. However, follow up of our cohort is currently underway and will allow the investigation of the relationship of cerebellar biochemistry with functional outcomes. Nonetheless, the overall relationships with advancing PMA prior to TEA and its alteration in presence of radiographic structural brain injury generates hypothesis for future studies investigating its pathophysiologic, diagnostic and prognostic significance.

## Conclusion

This study is the first to report an association between altered metabolic profiles prior to reaching TEA in the developing cerebellum with moderate-to-severe radiographic cerebellar brain injury. We report increasing trends in metabolite concentrations in the cerebellum with advancing PMA, which was altered in infants with brain injury on MRI. The relationship of metabolites with clinical complications may indicate a contributory role in etiopathogenesis of brain injury. Prospective studies of longitudinal cerebellar ^1^H-MRS with larger sample sizes and long-term neurodevelopmental follow-up are needed to better understand its pathophysiologic and prognostic value in guiding clinical interventions to improve outcomes.

## Data Availability

The datasets generated during and/or analyzed during the current study are available from the corresponding author on reasonable request.
